# The Relationship between Cyberchondria and Health Anxiety and the Moderating Role of Health Literacy among the Pakistani Public

**DOI:** 10.3390/ijerph21091168

**Published:** 2024-09-02

**Authors:** Preeda Sansakorn, Iqra Mushtaque, Muhammad Awais-E-Yazdan, Muhammad Khyzer Bin Dost

**Affiliations:** 1 Department of Occupational Health & Safety, School of Public Health, Walailak University, Tha Sala, Nakhon Si Thammarat 80161, Thailand; 2 Department of Psychology, University of Layyah, Layyah 31200, Pakistan; 3 Lahore Business School, University of Lahore, Lahore 54590, Pakistan

**Keywords:** cyberchondria, health anxiety, health literacy, developing country

## Abstract

Following the COVID-19 pandemic, the current study examines the association between cyberchondria and health anxiety in the Pakistani population, with health literacy as a moderator. This study utilized a cross-sectional research approach, with data gathered through simple random sampling. The study enlisted 1295 participants from Pakistan aged between 18 and 70, 63% of whom were male and 36% of whom were female. The researchers found a statistically significant positive link between cyberchondria and health anxiety (β = 0.215; t = 1.052; *p* 0.000). The moderating influence of health literacy suggests that health anxiety has a significantly negative effect on the relationship between cyberchondria and health anxiety (β = −0.769; t = 2.097; *p* 0.037). Moreover, females had higher cyberchondria scores than males. Health-related anxiety did not differ between the sexes, and males had greater health literacy than females. These results emphasize the critical role of health literacy in the moderating effects of cyberchondria on health anxiety. Furthermore, they reveal significant gender differences in both cyberchondria and health literacy.

## 1. Introduction

The word “cyberchondria”, a combination of “cyber” and “hypochondria”, describes a disorder in which a person’s health-related worry levels are raised by an obsessive obsession with searching out medical information online [1]. The spread of digital technology has made this syndrome more common. Cyberchondria sufferers worry too much about minor medical issues and experience illogical anxiety. Conversely, health anxiety is characterized by an unhealthy obsession with the risk of getting sick, which can impair a person’s capacity to go about their daily activities and appreciation of life in general [2].

Since its inception in December 2019, the COVID-19 pandemic has been associated with high morbidity and mortality rates, as well as physical and emotional implications, worldwide [1]. Studies on Pakistani students show that anxiety increased during the COVID-19 lockdown and quarantine period [3], which led to maladaptive behaviors, emotional distress, and avoidance in both the general public and patients [4]. Mental health is greatly affected by how psychological constructs, such as avoidance behavior, emotional suffering, and maladaptive behaviors, are interconnected. It is widely accepted that substance abuse and self-mutilation are unfavorable reactions to tough situations [5]. Distress caused by stressors can lead to extremely negative feelings, eventually resulting in mental disorders [6]. Avoidance behaviors refer to intentional disregard or escape from situations, ideas, or feelings that may result in annoyance or unease [7], and they can adversely affect an individual’s overall health status.

In Pakistan, both opportunities and challenges are brought about by the rapid growth of the internet. The internet offers useful information on health matters, but it is also dangerous, especially for those with cyberchondria [7]. This is made worse in the Pakistani context, where mental health remains a taboo subject of discussion. Instead of consulting a doctor, cyberchondriac individuals usually consult Google online, which perpetuates anxiety and spreads misconceptions. Misguidance from these internet-based sources may lead to incorrect diagnoses, heightened anxiety, and unnecessary visits to doctors, further straining the healthcare system in place. Furthermore, this situation is compounded by low levels of digital literacy within some sectors of society when they are unable to distinguish between reliable and unreliable sources.

This study makes a valuable contribution to the knowledge of cyberchondria, health anxiety, and the role of health literacy in Pakistan. It fills a research gap by providing figures outside of Western society, demonstrating regional cultural and societal dynamics. This study raises health literacy as an intervening factor, implying that improvement in health literacy would decrease health anxiety. Some practical applications are provided for healing interventions, such as instructive campaigns, reliable online sources of information on health issues, and feasible suggestions for enabling the merging of public policy with the process of health promotion through education. Thus, this article provides a starting point that can be used for future studies and also creates room for enhancing public health in Pakistan.

### 1.1. Literature Review

Psychological distress and anxiety disorders associated with widespread internet use are relatively new in psychiatric and medical settings [1]. The internet is a common source of health information for the public. According to Internet World Stats, the 4.93 billion people who use the internet globally reside in Asia (51.8%), Europe (14.8%), and Africa (12.8%). The global average for internet usage was 63.2%, indicating that the internet is a platform for reading, searching information, and reaching a large audience. Nearly half of 12,000 participants from 12 countries used Google for self-diagnosis [8]. An Indian English newspaper reported that 7% of all Google searches, or 70,000 queries per minute, are on health-related topics [8]. Repetitive media exposure to pandemic-related information and regular internet use lead to worsening of anxiety and the development of a pattern of psychological distress. This condition is known as cyberchondria [2,9].

People who access health information on the internet have become more concerned or distressed [10]. Illness anxiety, also known as illness anxiety disorder or health anxiety, causes patients to misinterpret minor body feelings or symptoms. They frequently consult healthcare professionals and request numerous medical tests, imagining worst-case scenarios about their health and how their health concerns could disrupt their daily lives and impair their functioning at work or their personal relationships. According to an indigenous study, 57.7% of people suffered from anxiety during the period of COVID-19 [11]. People with similar problems seeking medical advice online usually have cyberchondria [12]. People who suffer from high levels of health anxiety report longer and more frequent online searches on health-related topics, as well as higher levels of anxiety both during and after searches [13]. Fear is another reason why people seek health-related information [14].

Health-related reactions to epidemics (localized outbreaks that can affect cities) and pandemics (global outbreaks that affect multiple countries) can vary [15]. Understanding the causes and maintenance of anxiety can facilitate the development of preventive and treatment measures [16]. Health anxiety is a phenomenon with several aspects, ranging from a lack of health awareness to extreme health concerns or hypochondria [17]. According to cognitive–behavioral models of health anxiety and hypochondriasis, harmless physiological sensations are interpreted as distressing signs of serious illness, resulting in health anxiety and an increase in bodily symptoms [18]. Triggering events such as media reports can affect bodily sensations, perception, and interpretation. Certain predisposing factors (such as general anxiety) may make a vicious cycle of physical sensations, thoughts, and anxiety more likely, and illness behavior (such as safety-seeking behavior like internet research or visits to the doctor) may maintain the cycle via negative reinforcement [19,20]. During a virus outbreak, bodily sensations or symptoms may be interpreted as follows: “I’m breathing faster; I may have coronavirus”. In light of past pandemics (such as SARS), an overestimation of risk causes greater alarm [20].

Regarding use of the internet as a safety-seeking behavior, the media can have a significant impact on factors, such as excessive COVID-19-related information, which cause and sustain epidemics and pandemics [21]. Media use can be a safety-seeking behavior (e.g., studying viral symptoms) and can induce or promote more safety-seeking behavior (e.g., more or excessive internet use and clinic visits) [22]. Cyberchondria is a typical protection-seeking behavior and can amplify and/or sustain worries and fears [23]. During viral epidemics, individuals read more emotionally charged items in the media [24]. Moreover, Bergeron and Sanchez (2005) investigated the media coverage of the 2003 SARS outbreak in Norway and concluded that the media actively instilled anxiety in the public by showing only the most concerning examples or making incorrect comparisons [25]. Previous pandemics and the current COVID-19 pandemic have demonstrated that media coverage fosters panic [26]. There are inconsistencies between reports on the relationship between viral awareness and health anxiety [20]. Goulia et al. (2010) revealed that perceived knowledge reduces anxiety [27], and Blakey and Abramowitz (2017) discovered that viral knowledge increases anxiety [20]. In a study by Salari et al. (2020), there was no association between viral knowledge and anxiety; however, there was a correlation between wanting more information and reduced anxiety [28]. Only a few studies examined the link between media use, viral knowledge, and anxiety [21].

By improving health literacy, the current epidemic can be reduced [29]. Research has revealed that more than one-third of adults have difficulty accessing, digesting, analyzing, and utilizing health information [29]. People who lack health literacy are more likely to have a lower level of education, be older, or have a worse social status [30]. Health literacy may help to reduce the impact of COVID-19 stress on mental health. Frontline healthcare providers express exhaustion [31], anxiety, hopelessness, and post-traumatic stress disorder (PTSD). Techniques for coronavirus dissemination and containment may have an impact on the general public’s mental health [32]. According to previous studies, mental health has deteriorated in several countries since the outbreak. Only three studies [33,34,35] have examined the association between health literacy and mental health during the COVID-19 pandemic. The broad media and social media coverage of the pandemic, nicknamed the COVID-19 infodemic by some, may have a negative impact on low health literacy and poor mental health [36]. Because contradictory information is available, it is critical for individuals to carefully analyze information sources and discern between correct and erroneous information.

**Figure 1:**
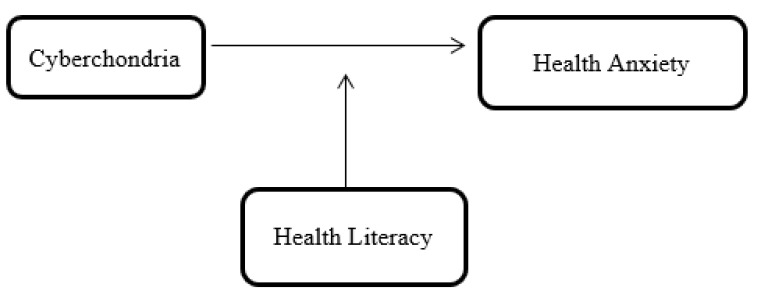
Conceptual framework of the study.

A novel idea that may differentiate cyberchondria from other anxiety disorders is that, from the perspective of a pandemic-related mental health crisis and massive deception, it appears crucial to pay attention to psychopathological phenomena. Cyberchondria is a type of health anxiety characterized by a compulsive need to obtain information from the internet, leading to unwarranted fears about one’s health. One distinguishing feature is the unwavering pursuit of medical knowledge, which frequently results in incorrect labeling of normal symptoms as severe medical diseases. Despite receiving reassurance, individuals with cyberchondria engage in constant monitoring of bodily symptoms, have a high frequency of clinic visits, and suffer from chronic anxiety. Despite a lack of empirical support, this concern stems from an established fear of developing life-threatening diseases. This harmful fixation severely hinders daily functioning and typically focuses on concerns that arise in the digital context. Therefore, this study examined the relationship between cyberchondria and health anxiety among the general public in Pakistan, using health literacy as a moderator (Figure 1). Research [33,34,35] suggests that an individual’s ability to manage health information enables them to make educated decisions, eliminate uncertainty, and engage in meaningful conversations with healthcare providers. Therefore, health literacy can help moderate the association between health anxiety and cyberchondria.

### 1.2. Hypotheses

**H1.**  *Cyberchondria has a significant association with health anxiety.*

**H2.**  *Health literacy moderates the relationship between cyberchondria and health anxiety.*

**H3.**  *There are variations between males and females in term of cyberchondria, health anxiety, and health literacy.*

## 2. Methodology

This study utilized a cross-sectional research approach, and a G power analysis was used to measure the sample size. The aforementioned computation was conducted based on a pre-established statistical significance level, accompanied by a power of 80%, a confidence level of 95%, and a margin of error established at 5%. The researchers determined that a sample size of 1295 persons was sufficient to accurately represent the population. The estimated sample size was 900, but we distributed 2000 questionnaires. Simple random sampling was used to select Pakistani adult representatives. The researchers approached colleges, public- and private-sector organizations, and rural areas for maximum participation of the general population. The participants provided informed consent, and data collection took place after the period of COVID-19, from July 2022 and January 2023. The study’s inclusion criteria were a minimum age of 18 years and no psychological illness, such as obsessive–compulsive disorder (OCD). After the demographic sheet, a screening OCD scale was attached, followed by the rest of the scales, i.e., the cyberchondria scale, the health anxiety scale, and the health literacy scale. A total of 240 participants refused to participate in the study; 375 forms were incomplete; and on the screen scale for OCD, we found that 90 patients who had mild to moderate symptoms of OCD were also excluded.

### 2.1. Tools for the Study

1. **Cyberchondria Severity Scale:** The Cyberchondria Severity Instrument—Short Form (CSS-12) is a 12-item self-report scale that is used to evaluate online health problems. Items are rated on a 5-point scale ranging from 1 (never) to 5 (always). Compulsion, discomfort, and medical skepticism were all measured on the scale. These findings indicate the presence of cyberchondria. In this study, the CSS-12 exhibited high internal consistency (Cronbach = 0.90) [37].
2. **Health Anxiety Scale:** The Short Health Anxiety Inventory is an 18-item self-report instrument that measures health anxiety over the past six months. The theory is founded on the cognitive model of health anxiety and hypochondria. (a) Health anxiety and the predicted probability of illness (14 items) and (b) anticipated adverse effects of the disease (4 items) are assessed [38].
3. **Health Literacy Scale:** The updated European Health Literacy Survey Questionnaire was used to assess health literacy (HLS19-Q12). The HLS19-Q12 questionnaire assesses an individual’s ability to find, comprehend, evaluate, and act on health information. It has been validated in Europe [39] and consists of 12 items that mimic the health literacy features specified by Sørensen et al. (2015) [40]. According to the European Health Literacy Survey, there are two levels of health literacy: insufficient health-related information (0–32) and sufficient health-related information (33–50).

### 2.2. Statistical Analysis

In total, we received 1295 completed responses, and analyses were applied to the data. In the current study, we used the descriptive statistic and partial least-squares structure equation model to check the variables’ association (PLS-SEM) with the gathered data. An independent-samples t-test was applied to measure gender-wise differences.

### 2.3. Ethical Committee Approval

This study was reviewed and approved by the Research Ethics Committee of the University of Layyah. An ethical review was conducted to ensure that the research complied with the ethical standards and guidelines set forth by the institutions. Informed consent was obtained from all participants, and confidentiality and privacy were maintained throughout the research process. 

## 3. Result

The current study included 1295 participants, with 825 (63%) males and 470 (37% females) participating (Table 1). The majority of participants were between the ages of 36 and 45. Furthermore, 54% of participants had completed high school. Medical history information was also collected from the study participants: 52% revealed that they had no disease at the time of filling out the questionnaire, while 47% had a medical history. Furthermore, 50% of the participants had an ailing family member in their home, and 44% admitted to looking up their family member’s symptoms on the internet practically every time they appeared. Based on the jobs of the people who participated in this study, there was a wide range of demographics: 23% of those surveyed worked as teachers, and 10.9% of the population worked as doctors or nurses. Out of all the people who answered, 14.4% worked in the law-and-order area, which was properly represented. There were 95 business owners in the sample, which is 7.3% of the total. In total, 5% of the people said they were farmers; 11% did not work. At last, 351 of the respondents were students which is 27.1% of whole population. In terms of where they lived, 875 (32.5%) of the respondents were from cities and 420 (32.5%) were from rural areas. Therefore, the data provided a full picture of the characteristics of the study population.

### 3.1. Assessment of Measurement Model

**Figure 2:**
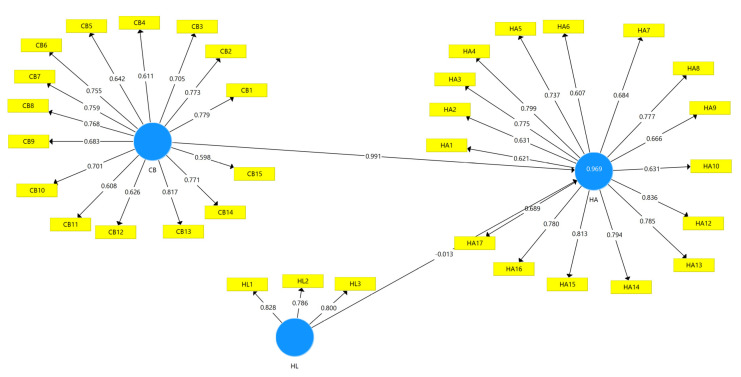
Measurement model. CB = cyberchondria, HA = health anxiety, HL = health literacy.

In this investigation, we used PLS-SEM as the data analysis method. Both the measurement and structural models were used in this study. Applying what is known as the outer or measurement model, we assessed the constructs’ convergent validity, discriminant validity, internal consistency reliability, and item reliability. The relevance of the route coefficient was assessed using an inner model (sometimes referred to as the structural model). The measurement model and its components are presented below (Figure 2).

### 3.2. Individual Items and Internal Consistency Reliability

The reliability of the items was assessed using the factor loadings of each construct [41]. According to Hair et al. (2014), it is advisable to retain items that have a burden ranging from 0.40 to 0.70 [42]. Items must be discarded if the composite reliability (CR) and average variance extracted (AVE) increase [42,43]. We eliminated only one item in this effort to increase the CR and AVE. The model maintained the remaining components. The reliability of internal consistency [42] assesses the degree to which the individual construct items predict the same construct. Internal consistency can be measured using composite reliability (CR) and Cronbach’s alpha [42]. Furthermore, Cronbach’s alpha and CR values are nearly indistinguishable; however, CR has gained widespread recognition [44] and has therefore been employed to assess the dependability of internal consistency. Between 0.70 and 0.90 is the acceptable range of values. All levels of internal consistency and reliability were acceptable (Table 2).

### 3.3. Convergent Validity

Convergent validity was measured using average variance extracted (AVE) [42]. By determining appropriate convergent validity, the AVE of each construct should be greater than 0.50. Table 2 shows that each construct’s AVE was greater than 0.50.

### 3.4. Discriminant Validity

Discriminant validity refers to the extent to which a certain construct can be distinguished from other constructs. This study employed three distinct methodologies to assess discriminant validity: the heterotrait–monotrait ratio of correlations (HTMT) [45], the Fornell–Larcker criterion (1981), and cross-loadings. The Fornell–Larcker criterion (1981) is employed to assess the discriminant validity of a construct using average variance extracted (AVE) values. Furthermore, in the context of cross-loadings, the factor loading of each construct should exceed that of other constructs [45]. Similarly, the HTMT statistic serves as a factor correlation measure that distinguishes between two factors [46]. Table 3 presents the Fornell–Larcker criterion, which assesses the discriminant validity of the variables in the current study. Table 4 displays the cross-loadings, which indicate the extent to which each variable loaded on its intended factor. Finally, Table 5 shows the heterotrait–monotrait ratio of the current study, which evaluates the convergent validity of the variables.

### 3.5. Assessment of Structural Model

**Figure 3:**
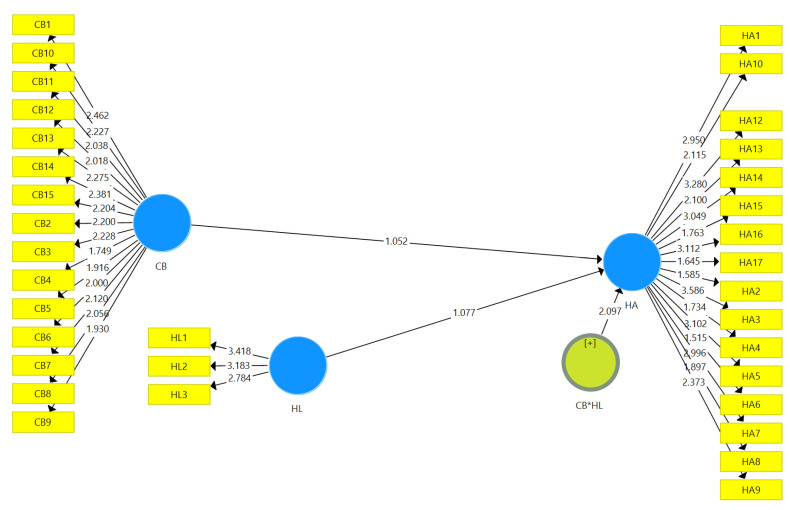
Structural model (direct relationships and moderating effects). CB*HL = moderating effect of health literacy between cyberchondria and health anxiety; CB = cyberchondria, HA = health anxiety, HL = health literacy.

To assess the structural model, we followed a standardized bootstrapping technique with 5000 bootstrap samples and 1295 samples to measure the significance of path coefficients. Figure 3 displays the measurements of the structural model (direct and moderating effects).

The structural model of the present study depicts the path coefficients of the hypothesized relationships. Hypothesis H1 states that cyberchondria has a significant association with health anxiety. The results in Figure 3 and Table 6 show a significant positive relationship between cyberchondria and health anxiety (β = 0.215; *t* = 1.052; *p* < 0.000). Similarly, hypothesis H2 states that health literacy negatively moderates the relationship between cyberchondria and health anxiety. The results in Figure 3 and Table 6 display the interaction effect, indicating that the effect of health anxiety on the association between cyberchondria and health anxiety (β = −0.769; *t* = 2.097; *p* < 0.037) is significantly negative.

**Figure 4:**
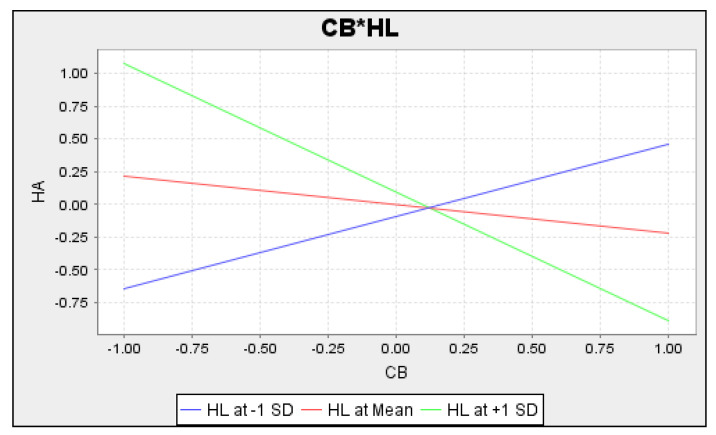
CB*HL = Interaction effect of health literacy between cyberchondria and health anxiety.

Figure 4 illustrates the representation of the independent and dependent variables along the *x*- and *y*-axes. The basic slope plot illustrates the relationship between the exogenous and endogenous constructs in the presence of varying moderator levels. This is represented by three distinct lines: green, red, and blue. The blue and green lines represent varying levels of the moderator, with blue indicating a low level and green indicating a high level. On the other hand, the red line illustrates the impact of the independent variable on the dependent variable when the moderating effect is not present.

Table 7 shows the gender-wise differences on the scale of cyberchondria, health anxiety, and health literacy among the selected sample of Pakistani individuals. The results revealed that on the cyberchondria scale, significant gender differences were found (t (1293) = −2.42 **, *p* = 0.003), as females had higher scores than males (mean female = 5.57 vs. mean male = 3.15). On the health-related anxiety scale, we did not find any statistical difference between males and females. Moreover, on the scale of health literacy, we found a statistical difference between males and females (t (1293) = 2.03 *, *p* = 0.02), as males had higher scores than females (mean male = 2.27 vs. mean female = 1.67).

## 4. Discussion

This study aimed to examine the association between cyberchondria and health anxiety among the Pakistani population and the moderating role of health literacy. According to Vismara et al. (2020), the amount of information on the prevalence of cyberchondria contained in published research is quite limited, particularly for the general population [12]. The results of the study and sample showed a positive association between cyberchondria and health anxiety in the general population (Table 6). Notably, the current study sample was a non-clinical sample. The results of Pakistani population sample-based research support our study’s results showing that metacognition and health anxiety significantly predict cyberchondria [47]. According to the current study results, 50% of cyberchondriacs look up their symptoms online (Table 1). Those who experienced moderate anxiety spent more time online than those who experienced mild anxiety. Their fear of disease was heightened by online medical consultations. Extreme sickness and anxiety exacerbate disability. Because they rarely find a single source of information sufficient, they frequently surf two or more websites simultaneously [48]. Physical symptoms include increased anxiety, a faster heartbeat, difficulty in breathing, and throat constriction. The longer a person is emotionally disturbed, the longer they spend looking for signs. They become progressively more convinced that they are ill as they hunt. A person with cyberchondria is more likely to trust the internet and distrust physicians. Massive online searches for health information may lead to self-medication [49]. Because the internet is the primary source of knowledge in the current era, health-conscious individuals and concerned family members will search online for logical causes of symptoms. People with excessive health anxiety misread their bodily symptoms and mistakenly believe that they are ill. People seek health assurance and relief from a variety of sources [50]. Online searches for health-related information can lead to unfavorable outcomes (e.g., increased anxiety and stress) and heighten health anxiety, which increases the frequency of such searches [13]. In a recent study, susceptibility to adult cyberchondria was investigated. According to one study [51], health concerns and low self-esteem led to increased internet health-related topic searches. People read internet health articles, blogs, and books to maintain their health and address health concerns. Only if the information comes from a reliable government source does health information on the internet make people with health anxiety more anxious than those without health anxiety. People who are already worried about their health are not made more worried by information from an internet community that is not as reliable [52]. As research has stated, social networking sites have significant negative effects on mental wellbeing [53].

The vast body of scientific research proving the relationship between health literacy and healthcare service use, disease self-management, preventative services, screening and immunization programs, and health-promoting activities attests to its importance [54]. In this study, we looked at health literacy as a moderating variable. The study’s findings show that Pakistanis have low health literacy, which is a significant contributing factor to public health anxiety. The majority of participants, being in secondary school, are not sufficiently qualified to understand medical conditions and terminology [55]. According to Tanis et al. (2016), people who are anxious about their health are less happy with medical consultations [56]. Cyberchondriacs could have an unfavorable view of doctors and do not believe that online health resources can replace consultations.

In the current study, we examined gender-wise differences in cyberchondria, health anxiety, and health literacy scales. We found that women had higher scores than men on cyberchondria scales. These results are consistent with those of a previous study that also found a high score for cyberchondria among the German general population [57]. Another study revealed that females had a higher mean cyberchondria score than males [58]. Our study found that on the scale of health anxiety, males and females have no statistical differences. Previous studies have revealed an association between health anxiety and sex [59]. Some studies have revealed that females have higher health anxiety than males [60]. However, some studies have revealed that males have higher health anxiety than females [61]. Only a few studies have found no gender differences on the scale of health anxiety [62,63,64]. Furthermore, our results revealed that men had a higher mean health literacy score than women did. A study revealed that women have poorer health literacy than men. This is because men are educated, have good reading skills, and have access to health-related information [65].

Despite its value, this study has some limitations that should be addressed by future researchers. First, secondary requirements exclude a significant number of prospective participants. Due to the fact that only individuals with at least intermediate qualifications were included in the sample, a large number of prospective volunteers were rejected. Regardless of educational attainment, researchers should consider adults. Future researchers should employ multi-method approaches, including questionnaires for self-reporting and formal interviews. Researchers should conduct interviews with participants to gain a greater understanding of their online health-information-seeking behavior. To validate these facts, participants’ relatives may be questioned. However, this research strategy will not resolve all the questions. In the future, longitudinal research should replace cross-sectional research.

### Implications for Management

Given the pervasive influence of the internet in modern society, it is highly unlikely that the frequency of online health inquiries will decrease. According to Starcevi and Berle (2013), to effectively address these concerns, it is recommended to adopt health literacy initiatives that provide individuals with guidance on the critical evaluation of health-related information [66]. One potential strategy for reducing the prevalence of inaccurate health information is the implementation of online consultations with healthcare professionals. Although there is currently no cure for cyberchondria, scientists have developed diagnostic methods. Cyberchondria is not listed in the DSM-5 or ICD-10 (ICD-11). Cyberchondria can be lessened though psychoeducation, and cognitive–behavioral therapy (CBT) has been found to be a potentially efficacious intervention for those suffering from significant health anxiety and cyberchondria. Patients diagnosed with cyberchondria must be made aware of its negative effects [67,68,69].

## 5. Conclusions

The study determined that a positive correlation exists between cyberchondria and health anxiety. Furthermore, the relationship between cyberchondria and health anxiety was negatively moderated by health literacy. Seeking information on the internet has been shown to increase health anxiety; due to lack of literacy in matters of health, people start believing the signs and this leads to higher health anxiety among the Pakistani population as a whole. We found that women had a higher score on the cyberchondria scale than men. Our study found that on the scale of health anxiety, males and females had no statistical differences. Furthermore, our results revealed that men had a higher mean health literacy score than women. A study revealed that women have poorer health literacy than men. Our findings also suggest that there is a need at the community level to educate people and increase their health literacy levels.

## Figures and Tables

**Table 1:** Demographic profile of the respondents.

Variable	F (%)
**Gender**	
Male	825 (63.7)
Female	470 (36.3)
**Participant Age Group**	
18–25	192 (14.9)
26–35	289 (22.3)
36–45	402 (31.1)
46–55	165 (12.7)
56–above 70	247 (19.1)
**Education Level**	
Secondary Level	583 (45.1)
Higher Level	712 (54.9)
**Medical History**	
Physical Disease	Yes 612 (47.2) No 683 (52.8)
**Occupation of Respondent**	
Teaching Profession	303 (23.3)
Medical Profession	142 (10.9)
Law-and-Order Profession	187 (14.4)
Own Business	95 (7.3)
Farming	65 (5.0)
Unemployed	152 (11.7)
Student	351 (27.1)
Residential Area	
Rural	420 (32.5)
Urban	875 (67.5)
**Do you have any patients at your home?**	Yes 655 (50.5) No 640 (49.5)
**How often do you look up your or your ill family member’s symptoms on the internet?**	Almost every time 571 (44.1) Never 255 (19.6) Occasionally 469 (36.2)

Notes: F = frequency, % = percentage.

**Table 2:** Loadings, composite reliability, and average variance extracted.

Constructs	Items	Loadings	CR	AVE
Cyberchondria	CB1	0.779	0.938	0.504
	CB2	0.773		
	CB3	0.705		
	CB4	0.611		
	CB5	0.642		
	CB6	0.755		
	CB7	0.759		
	CB8	0.768		
	CB9	0.683		
	CB10	0.701		
	CB11	0.608		
	CB12	0.626		
	CB13	0.817		
	CB14	0.771		
	CB15	0.598		
Health Anxiety	HA1	0.621	0.948	0.534
	HA2	0.631		
	HA3	0.775		
	HA4	0.799		
	HA5	0.737		
	HA6	0.607		
	HA7	0.684		
	HA8	0.777		
	HA9	0.666		
	HA10	0.631		
	HA12	0.836		
	HA13	0.785		
	HA14	0.794		
	HA15	0.813		
	HA16	0.780		
	HA17	0.689		
Health Literacy	HL1	0.828	0.846	0.647
	HL2	0.786		
	HL3	0.800		

CR = composite reliability, AVE = average variance extracted.

**Table 3:** Latent variable correlations and square roots of average variance extracted.

	CB	HA	HL
CB	**0.710**		
HA	0.685	**0.730**	
HL	0.518	0.500	**0.805**

Entries in boldface represent the square root of the average variance extracted; CB = cyberchondria, HA = health anxiety, HL = health literacy.

**Table 4:** Cross-loadings.

	CB	HA	HL
**CB1**	**0.779**	0.756	0.424
**CB2**	**0.773**	0.740	0.406
**CB3**	**0.705**	0.697	0.316
**CB4**	**0.611**	0.604	0.370
**CB5**	**0.642**	0.610	0.454
**CB6**	**0.755**	0.736	0.353
**CB7**	**0.759**	0.739	0.323
**CB8**	**0.768**	0.749	0.393
**CB9**	**0.683**	0.676	0.490
**CB10**	**0.701**	0.690	0.221
**CB11**	**0.608**	0.601	0.197
**CB12**	**0.626**	0.619	0.535
**CB13**	**0.817**	0.809	0.395
**CB14**	**0.771**	0.761	0.463
**CB15**	**0.598**	0.539	0.144
**HA1**	0.578	**0.621**	0.490
**HA2**	0.631	**0.649**	0.199
**HA3**	0.747	**0.775**	0.534
**HA4**	0.799	**0.819**	0.348
**HA5**	0.708	**0.737**	0.473
**HA6**	0.607	**0.619**	0.250
**HA7**	0.684	**0.689**	0.442
**HA8**	0.756	**0.777**	0.194
**HA9**	0.666	**0.686**	0.312
**HA10**	0.600	**0.631**	0.468
**HA12**	0.799	**0.836**	0.474
**HA13**	0.784	**0.785**	0.378
**HA14**	0.745	**0.794**	0.407
**HA15**	0.813	**0.814**	0.335
**HA16**	0.771	**0.780**	0.481
**HA17**	0.676	**0.689**	0.202
**HL1**	0.457	0.420	**0.828**
**HL2**	0.356	0.351	**0.786**
**HL3**	0.429	0.430	**0.800**

CB = cyberchondria, HA = health anxiety, HL = health literacy. Bold and highlighted values represents the loadings of each construct.

**Table 5:** HTMT correlation matrix for discriminant validity.

	CB	HA	HL
**CB**	-		
**HA**	0.469	-	
**HL**	0.624	0.601	-

**Table 6:** Structural model assessment with interactions.

Hypothesis	Relationships	Beta	SE	T-Value	*p*-Value	Decision
H1	CB->HA	0.215	0.205	1.052	0.000	Supported
H2	CB*HL->HA	−0.769	0.367	2.097	0.037	Supported

SE = standard error, CB*HL->HA = moderating effect of health literacy between cyberchondria and health anxiety.

**Table 7:** Gender-wise differences regarding scale of cyberchondria, health anxiety, and health literacy (N = 1295).

	Gender				
	Male	Female			
	(825)	(470)			
Scales	M (SD)	M (SD)	Sig.	t	df
**1. Cyberchondria**	3.15 (1.09)	5.57 (3.67)	0.003	−2.42 **	1293
**2. Health Anxiety**	1.96 (0.71)	2.01 (0.99)	0.53	1.75	1293
**3. Health Literacy**	2.27 (1.05)	1.67 (0.92)	0.02	2.03 *	1293

* Significance level 0.05, ** Significance level 0.01.

## Data Availability

The datasets used and/or analyzed during the current study are available from the corresponding author upon reasonable request.
